# AI-based decision models for difficult airway assessment: from research innovation to clinical implementation—a narrative review

**DOI:** 10.3389/fmed.2026.1818061

**Published:** 2026-05-13

**Authors:** Yang Shen, Yulan Wu, Yuwei Qiu, Jingxiang Wu

**Affiliations:** 1 School of Health Science and Engineering, University of Shanghai for Science and Technology, Shanghai, China; 2 Department of Anesthesiology, Shanghai Chest Hospital, Shanghai Jiao Tong University School of Medicine, Shanghai, China

**Keywords:** artificial intelligence, clinical prediction models, difficult airway, machine learning, perioperative medicine

## Abstract

Difficult airway management causes significant anesthesia-related morbidity, yet traditional assessments lack sensitivity (30%−50%) and consistency. This review (2010–2025) examines artificial intelligence (AI) decision models for airway assessment, focusing on performance, limitations, and clinical translation. AI demonstrates significant statistical superiority: facial image analysis achieves 80%−90% sensitivity (vs. Mallampati's 39%), and deep learning models yield a pooled AUC of 0.84. Key techniques include convolutional neural networks, semi-supervised learning, and multimodal integration. Despite high predictive performance, widespread adoption faces fundamental barriers. Current studies are predominantly single-center and retrospective, lacking external validation, algorithmic fairness, standardized outcomes, and proven workflow integration. Furthermore, research heavily favors upper airway evaluation. Thoracic anesthesia, utilizing routine preoperative CTs, offers an immediate pathway for comprehensive whole-airway assessment. Ultimately, bridging the translational gap requires rigorous, prospective multicenter validation demonstrating tangible patient safety improvements, rather than relying solely on algorithmic sophistication.

## Background

Unanticipated difficult airway management constitutes a critical perioperative patient safety concern, directly contributing to approximately 25% of anesthesia-related serious adverse events (1, 2). Despite decades of focus on safety protocols, the persistence of these events highlights a systemic failure in current predictive frameworks. Complications from failed intubation can rapidly induce life-threatening hypoxemia, leading to cardiac arrhythmias, hemodynamic collapse, and irreversible neurological injury within minutes (3, 4). Recent advances in artificial intelligence, particularly using images and clinical data, offer the potential to improve difficult airway prediction (5, 6), addressing a critical gap in current perioperative care. This underscores the paramount importance of accurate preoperative risk stratification for safe perioperative management.

Traditional preoperative assessment relies on established bedside examination techniques, yet these methods demonstrate well-documented limitations rooted in their inability to capture dynamic, three-dimensional airway anatomy. The poor prognostic value of the modified Mallampati score has been extensively documented (7), with the original clinical sign developed by Mallampati et al. (8) in 1985, subsequently modified (9, 10) despite persistent fundamental limitations. A comprehensive 2024 meta-analysis of 440 studies involving 686,089 patients confirmed consistently low sensitivity scores: Modified Mallampati test (0.39), thyromental distance (0.38), upper lip bite test (0.52), and Wilson risk score (0.42) (11). These findings align with earlier Cochrane systematic reviews showing sensitivity for predicting difficult laryngoscopy ranging from 0.22 to 0.67 (12). Large-scale real-world evidence from the Danish Anesthesia Database analysis of 188,064 patients further demonstrates that anesthesiologists' clinical predictions often fail to identify difficult airways preoperatively (13).

The physiological stress response induced by direct laryngoscopy—sympathetic activation, increased intracranial pressure, and altered airway tone—dynamically modifies anatomy in ways static assessments cannot capture. Moreover, the three-dimensional biomechanical interactions of pharyngeal and laryngeal structures during laryngoscopy exceed the analytical capacity of traditional bedside techniques.

The integration of artificial intelligence (AI) into perioperative care offers promising avenues to overcome these limitations through objective analysis of complex anatomical and physiological factors. This narrative review examines the research progress in AI-based decision models for difficult airway assessment, analyzing current developments, clinical performance, and implementation challenges specific to perioperative medicine.

## Definitions of difficult airway

“Difficult airway” is a broad clinical term with significant heterogeneity in its specific definitions across studies. The American Society of Anesthesiologists (ASA) guidelines classify it into seven types, including difficult mask ventilation, difficult laryngoscopy, and difficult tracheal intubation (14, 15). This review primarily focuses on the following key “difficult airway” endpoints.

### Difficult laryngoscopy (DL)

Refers to inadequate glottic visualization during laryngoscopy, commonly defined by Cormack–Lehane (C–L) grades III or IV (16). Early Mallampati classifications were used for prediction, but their positive predictive value has significantly decreased (9).

### Difficult intubation (DI)

A more complex definition, including multiple intubation attempts (e.g., >2 or 3) (13, 17, 18), or requiring advanced adjuncts (e.g., bougies, fiberoptic bronchoscopes) (16). Difficult intubation can occur even with a good laryngoscopic view.

### Difficult mask ventilation (DMV)

Defined as the inability to effectively maintain patient oxygenation during mask ventilation (e.g., SpO_2_ <92%) (2), potentially requiring a two-person technique or assistive devices (13, 19).

### Videolaryngoscopy view grades

Refers to glottic view assessment via videolaryngoscopy. The POGO score quantifies glottic exposure, reducing subjectivity, but its clinical adoption and effectiveness in differentiating nuanced difficulties are still being explored (20).

Acknowledging this definitional variability is crucial when appraising the generalizability and comparability of outcomes reported by different AI models, as inconsistent definitions can confound predictive performance evaluations across studies.

### Literature search strategy and methodological appraisal

**Figure 1:**
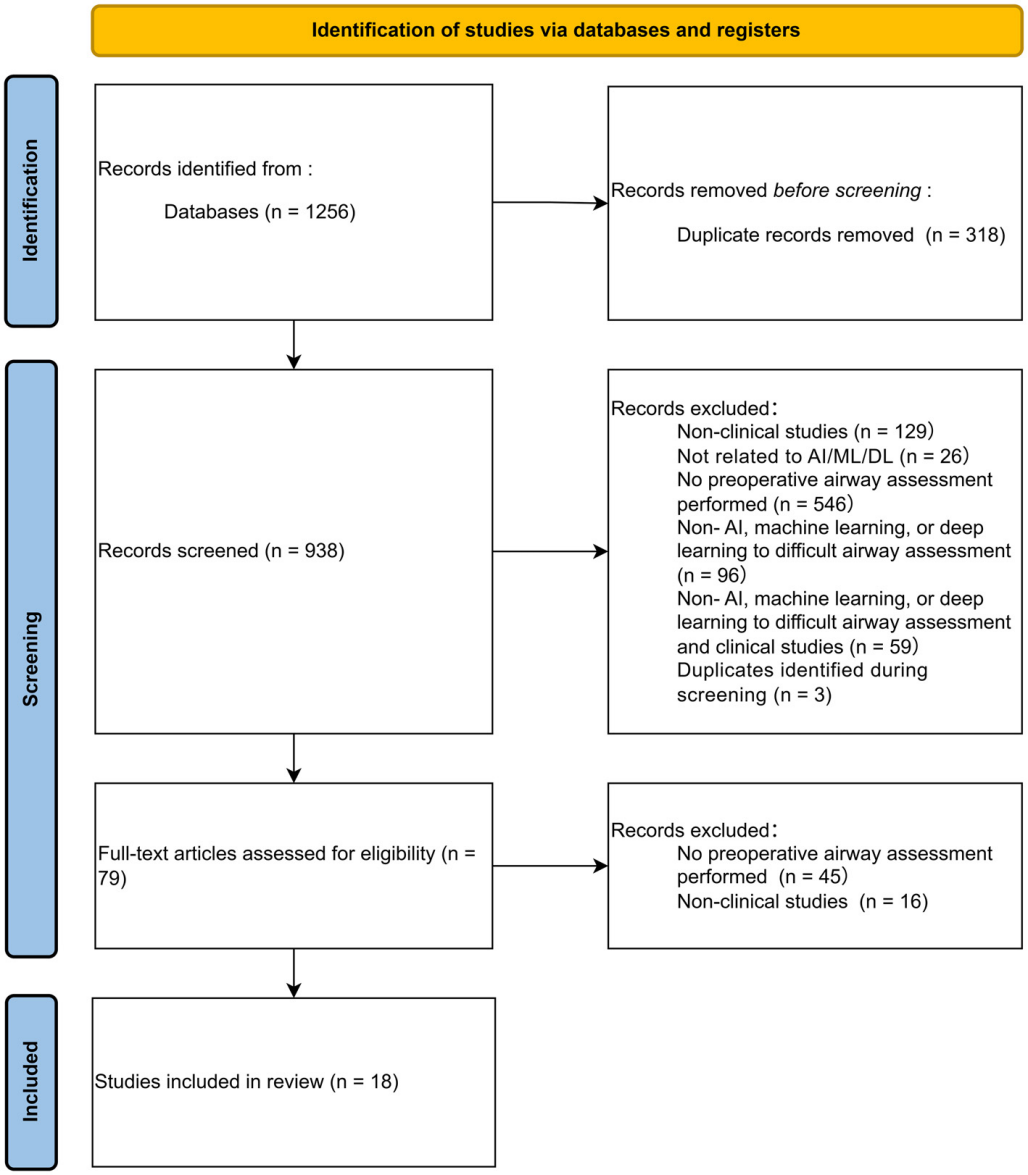
PRISMA Flow Diagram Detailing the Literature Search and Study Selection Process. This three-stage flow diagram illustrates the systematic process of identifying, screening, and including relevant studies for AI-based difficult airway assessment. The Identification stage aggregates initial literature from primary databases, including PubMed (*n* = 626), Embase (*n* = 355), and Scopus (*n* = 275), yielding 1,256 total records prior to the removal of 318 duplicates. The Screening stage processes the remaining 938 unique records by applying rigorous exclusion criteria to filter out inputs such as non-clinical studies, non-AI applications, and articles lacking preoperative airway assessments. A secondary full-text eligibility assessment further evaluates 79 articles, filtering out those without appropriate clinical focus. Finally, the Inclusion stage translates this comprehensive filtering process into a final curated cohort of 18 studies, all systematically integrated into the final review for critical synthesis and quality evaluation.

We conducted a systematic search in PubMed, Embase, and Scopus (January 2010–December 2025) using structured AI- and airway-related Boolean terms (full search strings in Supplementary File S1). Adhering to PRISMA guidelines (Figure 1), the initial 1,256 records underwent deduplication (*n* = 318). We screened 938 records by title/abstract and assessed 79 full-text articles for eligibility. Inclusion criteria required human clinical studies applying AI/machine learning to difficult airway assessment, reporting quantitative validations (e.g., AUC), sample size ≥20, and English full-text. Non-clinical studies and isolated abstracts were excluded. Ultimately, 18 original studies met all criteria for primary analysis. To ensure critical synthesis, the risk of bias and methodological quality of included models were evaluated using standard frameworks (Table 1).

**Table 1:** Representative AI studies in perioperative airway assessment.

Study and design	Dataset (*N*)	Clinical target and algorithm	Validation and optimization	Model performance	PROBAST risk
1. Arvind et al. (37) Dev	MC *N* = 4,087	Intubation (72h); RF	70/30 random split Class weight handling	AUC: 0.84 Calib: NR	Low
2. Çelik et al. (39) Dev	SC *N* = 1,486 → 341	LIII/IV; RF	10-fold CV	Sensitivity: 92.85% Calib: NR	High
3. Cho et al. (46) Dev + Val	SC *N* = 5,939	LIII/IV; 4-layer CNN	10 × random splits Bal. loss	AUC: 0.965 Calib: Yes (Brier: 0.023)	Low
4. Demir Senoglu et al. (31) Dev	SC *N* = 329	LIII/IV; SVM	80/20 train/test + CV SMOTE (train)	AUC: 0.852 Calib: NR	High
5. García-García et al. (18) Dev	MC *N* = 623	Comp. endp XGB	Nested 10-fold CV MICE and BL-SMOTE	AUC: 0.746–0.766 Calib: Yes (Plot)	Low
6. Hayasaka et al. (35) Dev	SC *N* = 202	LIII/IV; VGG16 + TL	Fold CV Aug and oversampling	AUC: 0.864 Calib: NR	Low
7. Kim et al. (45) Dev	SC *N* = 37,057	ETT size/depth GBRT	80/20 temporal split BorutaSHAP and MICE	F1: 0.502–0.669 Calib: NR	Low
8. Kim et al. (36) Dev	SC *N* = 616	LIII/IV; LGBM	80/20 random split	AUC: 0.710 Calib: NR	High
9. Kim et al. (20) Dev	SC *N* = 212	POGO score (Regr.) Ridge/Lasso	80/20 random split	R^2^: 0.12–0.21 Calib: NR	High
10. Kim et al. (33) Dev	SC *N* = 1283^*^	LIII/IV; EffNet-B5	Fold CV Mult.	AUC: 0.81–0.88 Calib: NR	High
11. Kim et al. (34) Dev + Val	SC *N* = 1,677	LIII/IV; Bal. RF	80/20 random split	AUC: 0.790 Calib: NR	High
12. Ming et al. (44) Dev	SC *N* = 390	L tube size (6 classes) ERNIE	70/30 random split	Accuracy: 0.770 Calib: NR	High
13.Pei et al. (30) Dev + Val	SC *N* = 669	Diff. mask vent.; LR + PCA	Fold CV PCA dim rec.	AUC: 0.825 Calib: NR	Low
14. Rodiera et al. (40) Dev	MC *N* = 313	LIII/IV; LR.	70/30 random split	AUC: 0.90–0.91 Calib: NR	High
15. Tavolara et al. (32) Dev	SC *N* = 152	Diff. Int. CNN Ens.	Fold CV Attention-based MIL	AUC: 0.710 Calib: NR	High
16. Xia et al. (29) Dev + Val	SC *N* = 5,849	Diff. Vide.; ResNet-18 + LGBM	60/20/20 split Focal loss/Aug	AUC: 0.779 Calib: Yes (Brier)	Low
17. Yan et al. (38) Dev + Val	Tr. & Ext. Val *N* = 247 + 82	LIII/IV; LASSO + LR	Temporal Ex-Val 2018–2020 vs. 2021)	AUC: 0.970 Calib: Yes (Plot/Brier)	Low
18. Zhou et al. (27) Dev	SC *N* = 500	Comp. Endp; GB	70/30 random split (Retro)	AUC: 0.848 Calib: NR	High

MC, multicenter; SC, single-center; Dev, development; Val, validation; Ext. Val, external validation; Tr., training; LIII/IV, Cormack–Lehane III/IV; Comp. endp., composite endpoint; XGB, XGBoost; TL, transfer learning; LGBM, LightGBM; EffNet-B5, EfficientNet-B5; Bal. RF, balanced RF; ERNIE, Baidu ERNIE; Diff. mask vent., difficult mask ventilation; LR, logistic regression; Diff. Int., difficult intubation; CNN Ens., CNN ensemble; Diff. Vide., difficult videolaryngoscopy; GB, gradient booting; Bal. loss, balanced loss approach; SMOTE (train), SMOTE on train set; BL-SMOTE, borderline-SMOTE; Aug, augmentation; Mult., multitask learning; dim rec, dimensionality reduction; Attn-based MIL, attention-based multiple instance learning; Retro, retrospective; Calib, calibration; NR, not reported. ^*^Undersampling.

## Limitations of traditional preoperative assessment

Traditional difficult airway assessment methods demonstrate fundamental limitations that compromise perioperative safety. The Mallampati classification, despite being the most widely used preoperative screening tool, achieves only 39% sensitivity in recent meta-analyses (11). This poor performance stems from its static evaluation approach that fails to account for dynamic three-dimensional interactions of pharyngeal and laryngeal structures during laryngoscopy (7).

Inter-observer variability compounds these limitations significantly, with studies demonstrating only moderate agreement between experienced clinicians when applying traditional assessment methods. This variability reflects the subjective nature of visual assessment and the difficulty in standardizing evaluation techniques across different clinical environments.

Composite scoring systems attempt to improve accuracy through multivariate approaches. The Wilson risk score integrates five clinical parameters, achieving a positive likelihood ratio of 10.9 when using a risk-sum criterion of ≥3 (21). The LEMON criteria enable systematic airway assessment and effective risk stratification for difficult intubation in emergency settings, though they exhibit inherent limitations of low specificity, as demonstrated by their inability to exclude false-positive predictions (22). These limitations persist because traditional methods cannot capture critical factors such as soft tissue compliance, tongue base volume, and epiglottic morphology, which are crucial determinants of laryngoscopic success.

Trends in preoperative airway assessment demonstrate significant institutional variations (23). Advanced imaging techniques mark progress toward objectivity, with CT-based anterior neck soft tissue measurements predicting difficult laryngoscopy with 75% sensitivity and 93.8% specificity (24). Ganjoo et al. (25) demonstrated that CT-derived measurements significantly optimized endotracheal tube size selection in 47% of head and neck cancer patients and reduced intraoperative external laryngeal manipulation by 75%. Barclay-Steuart et al. (26) developed comprehensive scoring systems using transnasal videoendoscopy for preoperative airway risk stratification, achieving AUC of 0.74 when combined with traditional parameters (26). However, these approaches require additional imaging not routinely performed for most surgical patients, limiting widespread implementation in standard perioperative care.

Building on these imaging advances, early machine learning applications established foundational evidence for computational superiority, demonstrating that algorithmic approaches could outperform traditional clinical assessment in specific populations. Zhou et al. (27) achieved an AUC of 0.848 in predicting difficult intubation for thyroid surgery using the Gradient Boosting algorithm, while Yamanaka et al. (28) demonstrated feasibility in emergency department settings.

The fundamental challenge facing traditional assessment methods is their inability to process complex, multidimensional anatomical relationships that determine airway difficulty. Practice variations across institutions further complicate standardization efforts, creating heterogeneity that limits universally applicable prediction models. This limitation creates an urgent need for more sophisticated analytical approaches capable of integrating multiple variables objectively.

### AI-powered perioperative risk stratification

**Figure 2:**
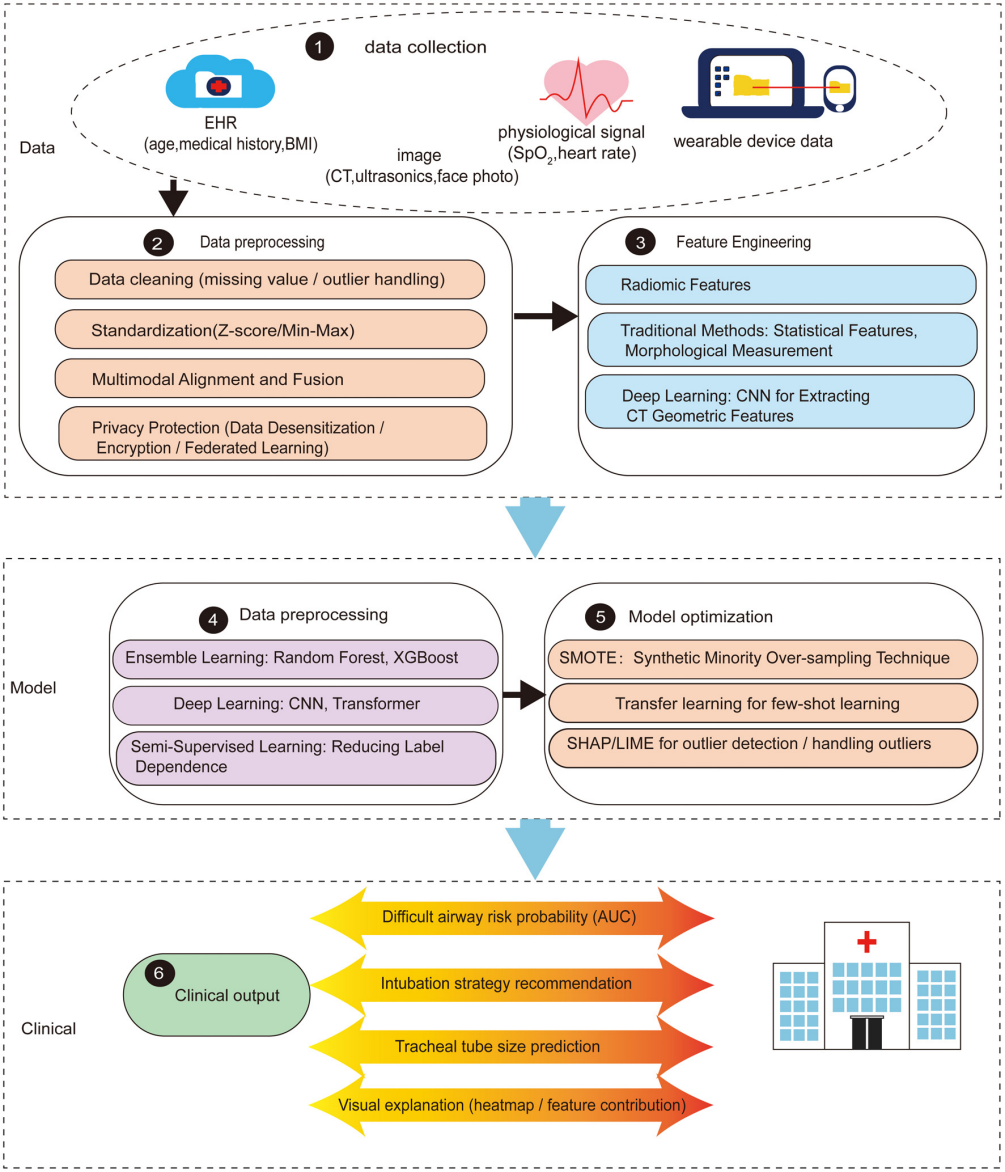
Technical workflow for ai-assisted perioperative airway assessment. This figure outlines the comprehensive workflow from data acquisition to clinical deployment. Step 1 involves data collection from multiple sources. Step 2 encompasses data preprocessing and feature extraction from electronic health records (age, medical history, BMI), imaging modalities (CT, ultrasound, facial photographs), and physiological signals (SpO_2_, heart rate). Step 3 focuses on feature engineerwing. Step 4 implements diverse AI architectures, including ensemble learning (Random Forest, XGBoost), deep learning (CNN, Transformer), and semi-supervised approaches to reduce labeling requirements. Step 5 incorporates model optimization techniques: SMOTE for handling class imbalance, transfer learning for few-shot scenarios, and SHAP/LIME for model interpretability and outlier analysis. Step 6 translates model outputs into clinical applications, including difficult airway risk probability (with AUC validation), intubation strategy recommendations, tracheal tube size prediction, and visual explanations (heatmaps, feature contributions) to support perioperative decision-making.

Artificial intelligence approaches offer transformative potential for preoperative airway risk stratification through sophisticated pattern recognition and multimodal data integration capabilities that surpass traditional clinical assessment methods. However, a critical appraisal of the current literature reveals both significant algorithmic advancements and persistent methodological challenges. The standard technical pipeline encompassing this data-to-decision methodology is detailed in Figure 2.

### Computer vision and morphometric deep learning

Facial image analysis using convolutional neural networks (CNNs) represents a core component of recent AI airway assessments. Using facial photographs, deep learning-based facial analysis has been utilized to efficiently predict difficult clinical scenarios, generating visualized class activation heatmaps that focus on clinically relevant anatomical regions like the chin and neck (29). Building upon this, recent studies have addressed the lack of volumetric data in 2D imaging. For instance, the application of geometric morphometrics combined with machine learning to 3D facial scans has successfully captured complex spatial morphological changes to accurately predict difficult mask ventilation (30). While promising, the external validity of these imaging models often remains contingent on the racial and anatomical diversity of their training datasets. These morphological evaluations are further strengthened by integrating algorithmic processing with traditional measurements of neck and laryngeal alignment (31).

To improve robustness in real-world clinical environments, researchers are evolving toward multi-model and simplified pathways. Recent advancements include the identification of difficult-to-intubate patients from frontal face images using an ensemble of deep learning models, which significantly outperforms individual models and enhances baseline predictive power (32). Furthermore, a growing trend prioritizes clinical feasibility; recent prospective studies have demonstrated the high efficacy of using deep learning models—which integrate multiple anatomical features—paired with minimal image analysis to significantly improve difficult direct laryngoscopy prediction directly at the bedside (33), representing a crucial shift from theoretical statistical accuracy to practical clinical utility.

### Clinical parameter-based and real-time decision support

Beyond imaging, the integration of objective patient measurements presents opportunities for real-time risk stratification. Machine learning predictions have been successfully applied to video laryngoscopy views, identifying key clinical predictors for safer intubation during the perioperative process (20). To improve precision in high-risk cohorts, specialized algorithms have been deeply validated. For example, prediction models leveraging simple, non-invasive measurements—specifically neck circumference and thyromental height—have demonstrated substantial clinical value for estimating difficult laryngoscopy (34). Similarly, tailored multi-algorithm predictive models have achieved high accuracy in predicting difficult airway intubation specifically for patients undergoing thyroid surgery, addressing the complex airway distortions typically caused by neck masses (27). Nevertheless, the performance of such highly specialized algorithms must be cautiously interpreted, as models trained on specific pathologies (e.g., thyroid masses) inherently risk overfitting and may lack generalizability across broader, unselected surgical populations.

### Clinical validation and retrospective cohort evidence

The clinical significance of computing methodologies is most apparent in large-scale cohort validations, though these rely heavily on the quality, standardization, and completeness of historical data. To replace subjective clinical grading, extensive research has focused on the broader creation of artificial intelligence models based on routinely collected parameters (35). Specifically, predictive models for difficult laryngoscopy utilizing machine learning via retrospective cohort studies have established a strong foundation for integrating AI into preoperative workflows (36). Through diverse variables, these systems enable the reliable prediction of a difficult airway for tracheal intubation extracted entirely from patient baseline electronic health records (18). Consequently, the successful development of various machine learning algorithms to predict intubation demonstrates that multimodal data can yield exceptionally high discriminative power (37), establishing robust framework methodologies for a prediction model for difficult intubation that consistently outperforms traditional logistic regression (38). Despite these statistical successes, true clinical validation requires rigorous, prospective, multi-center trials. Currently, while the reliable prediction of difficult tracheal intubation by artificial intelligence clearly illustrates its potential to significantly reduce adverse respiratory events across diverse surgical populations (39), the transition from retrospective statistical superiority to proven, real-world clinical benefit remains a pivotal challenge that future studies must address.

## Perioperative workflow integration challenges

Several categories of barriers must be addressed before AI-based airway assessment can be integrated into routine perioperative care.

### Methodological limitations and bias risks

A critical appraisal of currently included literature highlights prevailing methodological barriers to translating technical success into routine care. While innovative non-visual modalities have been explored—such as preoperatively predicting a difficult airway based on machine learning algorithms applied to patient voice analysis—these approaches emphasize how ambient noise interference and smaller validation cohorts can challenge predictive reliability (40). Furthermore, many evaluated models in current practice remain single-center or strictly retrospective investigations. This fundamental limitation elevates the risk of algorithmic overfitting and significantly hinders generalized application across diverse perioperative environments and varying demographic factors.

### Technical integration and workflow disruption

**Figure 3:**
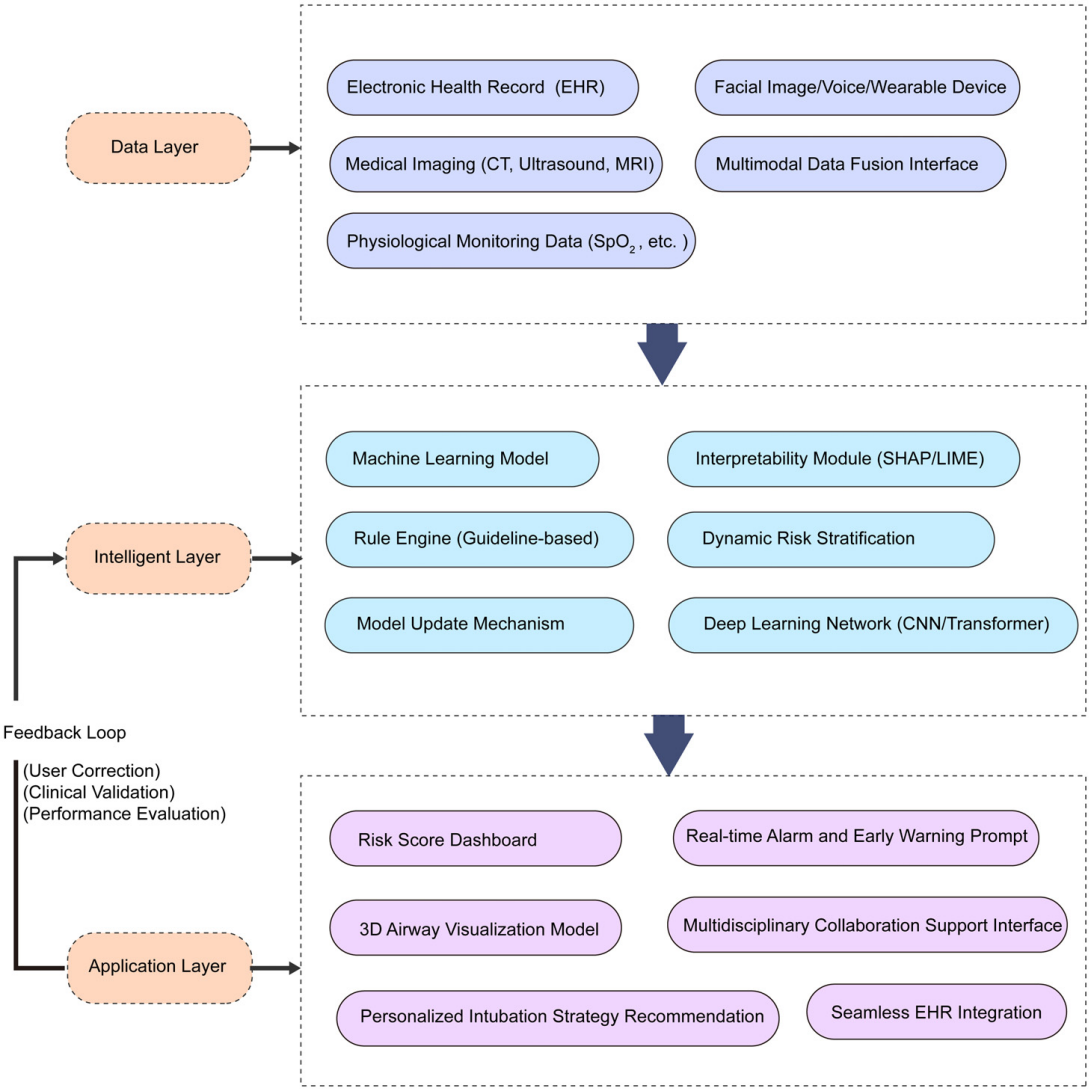
AI-based difficult airway assessment framework for perioperative care. This three-layer architecture illustrates the integration of artificial intelligence in perioperative airway assessment. The Data Layer aggregates multimodal patient data, including electronic health records (EHR), medical imaging (CT, ultrasound, MRI), facial images, and physiological monitoring (SpO_2_, heart rate). The Intelligent Layer processes these inputs through machine learning and deep learning models (CNN, Transformer), enhanced by interpretability modules (SHAP/LIME) and rule-based guidelines to generate dynamic risk stratification. A feedback loop incorporating user correction, clinical validation, and performance evaluation enables continuous model optimization. The Application Layer translates outputs into clinical tools including risk score dashboards, real-time alarms, 3D airway visualization, personalized intubation strategies, and multidisciplinary collaboration interfaces, all seamlessly integrated with existing EHR systems.

Figure 3 outlines a conceptual framework for integrating AI into perioperative workflows, from data acquisition to clinical deployment. However, translating this framework into practice presents significant technical and organizational challenges. Elhaddad and Hamam (41) examined AI-driven clinical decision support systems, identifying key barriers including workflow integration requirements, real-time processing capabilities, and interoperability with existing anesthesia information management systems. Successful implementation requires seamless integration without disrupting established perioperative practices or significantly increasing procedure time.

Moreover, observed variability in airway assessment trends impacts model development and generalizability across different surgical environments. representing a fundamental challenge requiring urgent standardization of data collection and processing protocols.

### Clinical acceptance and regulatory considerations

Clinical acceptance among perioperative providers depends heavily on system reliability, interpretability, and demonstrated clinical value rather than technical sophistication alone. For instance, while tools like ChatGPT may optimize clinical decision support (42), the “black box” nature of many Deep Learning systems remains a significant barrier. Anesthesia providers prioritize high “explainability” (e.g., why a patient is flagged as high-risk) for safe perioperative care, yet few current studies integrate SHAP or LIME-based interpretability modules into their final clinical interface.

Regarding regulatory frameworks, Vasey et al. (43) developed DECIDE-AI reporting guidelines for early-stage clinical evaluation. However, perioperative AI systems require careful navigation of safety and efficacy requirements through appropriate clinical trials and post-market surveillance protocols.

## Implementation opportunities in specialized care

Despite these challenges, certain perioperative settings offer immediate opportunities for AI implementation.

### Thoracic anesthesia: immediate implementation pathway

While the broader implementation of AI in perioperative airway management faces significant hurdles, thoracic anesthesia presents a particularly promising and immediate pathway for initial deployment. This is primarily due to the routine availability of preoperative chest CT imaging and the inherently complex airway management requirements often encountered in thoracic surgical patients.

### Rationale for thoracic anesthesia as an implementation pathway

While general anesthesia currently faces broad validation challenges, thoracic airway management presents a highly actionable pathway for AI implementation. Thoracic procedures frequently require precise endotracheal placement. Utilizing routine preoperative imaging, recent AI frameworks based on radiomics have been established as highly predictive models for identifying exact tracheal tube sizes in adult double-lumen endotracheal intubation (44). This concept extends directly into broader clinical goals of effectively predicting the optimal endotracheal tube dimensions to minimize the risk of airway trauma (45). Furthermore, deep-learning models associating lateral cervical radiographs with critical morphological structures have successfully automated the quantification of airway geometry before anesthesia induction (46), bypassing the subjectivity of traditional manual evaluations.

### Potential limitations and considerations

Despite these advantages, it is crucial to acknowledge that even within thoracic anesthesia, the implementation of AI is not without its challenges. These include the need for specialized AI models trained specifically on thoracic CT data, ensuring interoperability with existing hospital information systems, and addressing the regulatory and ethical considerations associated with AI in high-stakes clinical decision-making. Furthermore, while CT provides excellent anatomical detail, it does not capture dynamic airway changes during laryngoscopy or intubation. Therefore, a multimodal approach integrating CT data with other clinical and physiological parameters would likely be most effective. Realizing this vision will require dedicated research, multicenter validation, and a clear demonstration of improved patient safety outcomes in this specialized population before broader adoption.

## Emergency and critical care applications

Emergency perioperative scenarios face unique challenges, including time constraints, limited data availability, and high-stakes decision-making requirements. Mosier et al. (47) described the physiologically difficult airway, while Karamchandani et al. (48) outlined emergency airway management outside the operating room. AI systems for these environments must provide rapid, reliable assessments with clear uncertainty quantification and minimal data input requirements while maintaining high accuracy under pressure.

## Future directions for perioperative medicine

Looking forward, several research priorities emerge from this analysis to bridge the current translational gap.

### Multicenter validation, algorithmic fairness, and evidence generation

Future research must move beyond retrospective *in-silico* testing and prioritize rigorous, prospective external validation with standardized protocols. Crucially, models must be evaluated for temporal degradation and algorithmic fairness across distinct demographic groups to ensure equitable perioperative care. Ji et al. (49) developed evaluation frameworks for successful artificial intelligence-enabled clinical decision support systems, providing methodological foundations for validation. International collaborative networks could address current validation gaps and establish robust evidence bases through prospective randomized controlled trials (RCTs), which remain the gold standard for clinical implementation across diverse healthcare systems.

### Emerging technologies, LLMs, and interpretability risks

Foundation models and large language models present emerging opportunities for comprehensive perioperative assessment platforms. Thirunavukarasu et al. (50) demonstrated large language models in medicine, suggesting their applicability for integrated systems combining multiple data sources, including clinical notes, imaging reports, and structured assessment data. However, the deployment of LLMs in high-stakes perioperative environments introduces profound risks, including algorithmic hallucinations, lack of deterministic output, and complex patient data privacy concerns. As these technologies advance, parallel efforts have emerged to establish reporting standards that ensure methodological rigor and clinical applicability. Building on the TRIPOD+AI framework for clinical prediction models (51), Gallifant et al. (52) introduced the TRIPOD-LLM guidelines, which must be strictly adhered to by future researchers to mitigate the inherent “black-box” vulnerabilities of these advanced models.

## Implementation science and real-world performance

Future research should examine practical implementation strategies and real-world performance in diverse perioperative environments. Sendak et al. (53) explored presenting machine learning model information to clinical end users, while Shah et al. (54) addressed making machine learning models clinically useful. Understanding barriers to adoption and developing evidence-based implementation strategies is crucial for successful clinical translation beyond research settings. Gupta et al. (55) provided insights into AI-driven decision support for acute respiratory failure, demonstrating broader applications in critical care settings that could inform airway management approaches.

## Conclusions

This review demonstrates that AI-based models, particularly those using facial image analysis and multimodal data integration, have significant potential to overcome the limitations of traditional preoperative airway assessment methods. Current evidence from 47,044 patients across multiple studies confirms AI's statistical superiority, with deep learning models achieving AUC values of 0.84 compared to traditional methods with sensitivity below 50%. Nevertheless, this quantitative superiority has not yet definitively translated into proven, generalizable clinical utility. The path to perioperative clinical adoption requires addressing critical methodological challenges, including rigorous external validation, standardization of outcome definitions, and the unproven efficacy of seamless integration into existing perioperative workflows.

Thoracic anesthesia presents an immediate opportunity for clinical implementation due to routine preoperative CT imaging availability and complex airway management requirements that align well with AI capabilities. The specialized nature of thoracic surgery provides a controlled environment for initial deployment while generating evidence for broader perioperative applications.

Future research priorities must focus on demonstrating improvements in patient safety outcomes—the ultimate measure of success for any perioperative intervention. This necessitates moving beyond isolated predictive performance metrics (such as AUC and sensitivity) to robustly evaluate the impact of AI integration on clinical endpoints such as reduced morbidity, mortality, and improved resource utilization. The evolutionary integration of AI-powered decision support systems should augment—not replace—clinical expertise, ultimately enhancing perioperative airway management safety through evidence-based technological advancement.
