# Supplementary material for: AI-based decision models for difficult airway assessment: from research innovation to clinical implementation—a narrative review
Source: Front Med (Lausanne). 2026 May 13;13:1818061. doi: 10.3389/fmed.2026.1818061 (PMC13212430; doi:10.3389/fmed.2026.1818061)

**Search Strategy:**

PubMed Search Strategy:

1."artificial intelligence"[MeSH Terms] OR "artificial intelligence"[Title/Abstract]

2."machine learning"[Title/Abstract] OR "deep learning"[Title/Abstract]

3."neural networks"[MeSH Terms] OR "convolutional neural network"[Title/Abstract]

4."computer vision"[Title/Abstract] OR "image processing"[Title/Abstract]

5.#1 OR #2 OR #3 OR #4

6."difficult airway"[Title/Abstract] OR "airway assessment"[Title/Abstract]

7."intubation difficulty"[Title/Abstract] OR "laryngoscopy"[Title/Abstract]

8."airway management"[MeSH Terms] OR "airway management"[Title/Abstract]

9."mallampati"[Title/Abstract] OR "cormack lehane"[Title/Abstract]

10.6 OR #7 OR #8 OR #9

11.#5 AND #10

12.Filters: Humans, English, 2010-2025


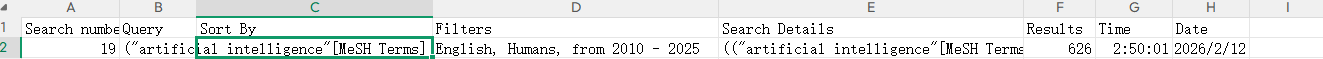


Embase Search Strategy:

1.'artificial intelligence'/exp OR 'artificial intelligence':ti,ab

2.'machine learning':ti,ab OR 'deep learning':ti,ab

3.'neural network'/exp OR 'convolutional neural network':ti,ab

4.'computer vision':ti,ab OR 'image processing':ti,ab

5.#1 OR #2 OR #3 OR #4

6.'difficult airway':ti,ab OR 'airway assessment':ti,ab

7.'intubation difficulty':ti,ab OR 'laryngoscopy'/exp

8.'airway management'/exp OR 'airway management':ti,ab

9.'mallampati':ti,ab OR 'cormack lehane':ti,ab

10.#6 OR #7 OR #8 OR #9

11.#5 AND #10

12.Limit to: human, English, 2010-2025


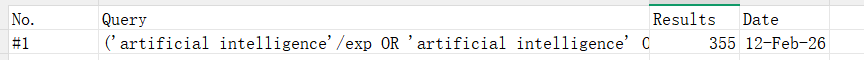


Scopus Search Strategy:

(TITLE-ABS-KEY("artificial intelligence" OR "machine learning" OR "deep learning" OR "neural network*" OR "computer vision"))

AND

(TITLE-ABS-KEY("difficult airway" OR "airway assessment" OR "intubation difficulty" OR "laryngoscopy" OR "airway management" OR "Mallampati"))

AND

(PUBYEAR > 2009 AND PUBYEAR < 2026)

AND

(LIMIT-TO(LANGUAGE, "English"))

AND

(LIMIT-TO(SRCTYPE, "j"))

AND

(LIMIT-TO(EXACTKEYWORD, "Human"))
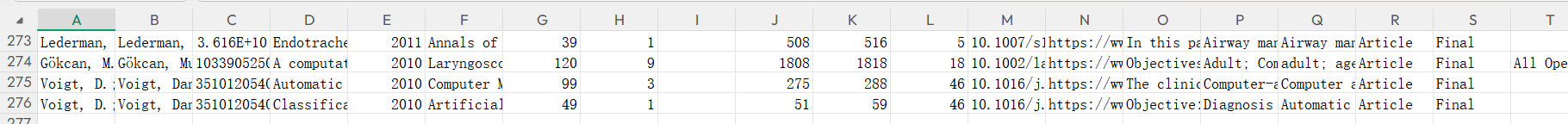


**Eligibility Criteria:**

Inclusion:

1.Studies applying AI, machine learning, or deep learning to difficult airway assessment

2.Clinical studies with human participants

3.Studies reporting validation data or performance metrics

4.Full-text articles available

Exclusion:

1.Conference abstracts only

2.Non-clinical studies (animal/simulation only)

3.Sample size <20 patients

4.Studies without AI/ML components

5.Non-English articles

**N-rank results：**


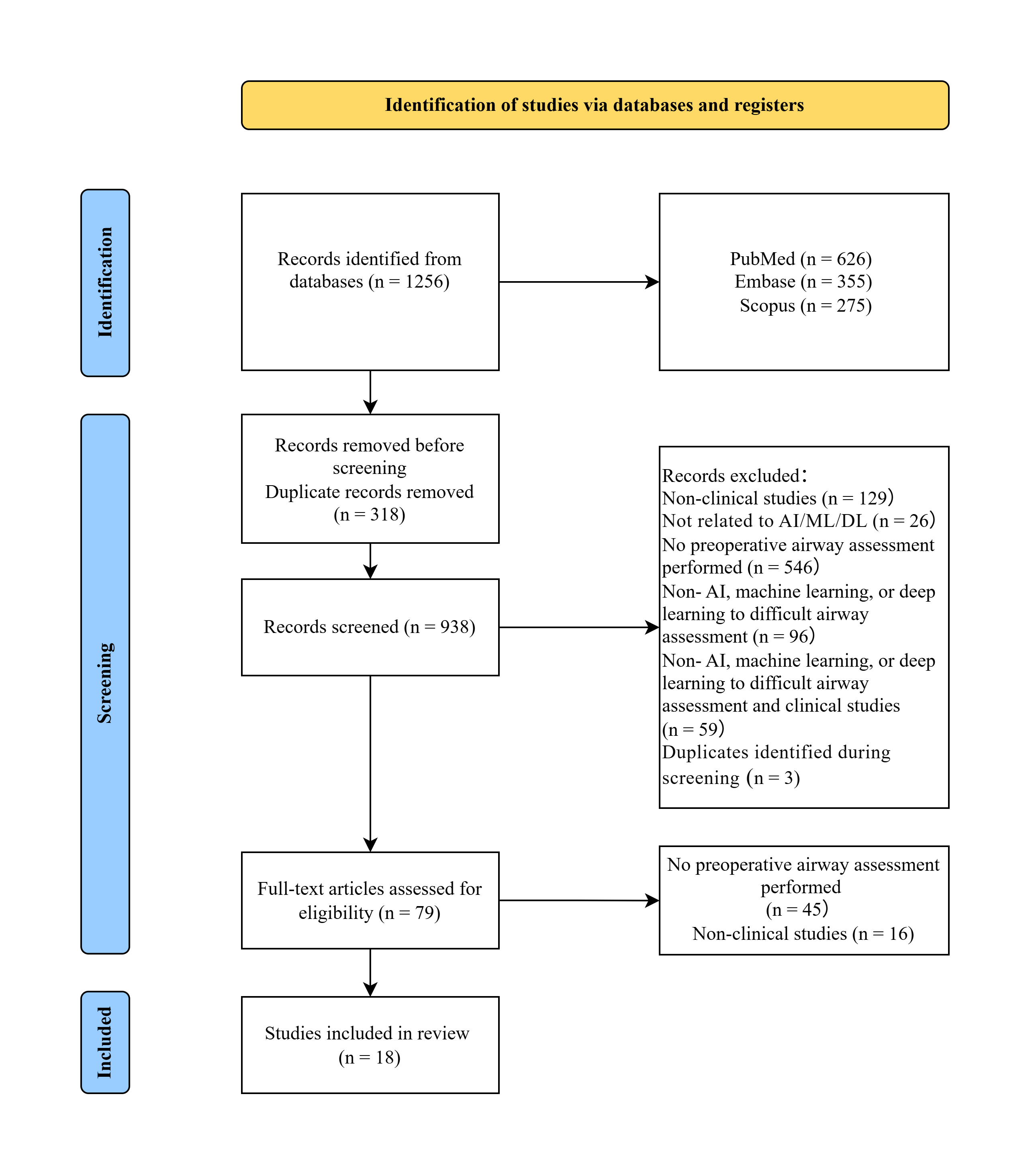

Supplement: Supplementary file 1 [file Table_1.docx]
